# Supplementary material for: High-Ratio Partial Proteolysis with Carrier Proteome (HOLSER) Enables Global Structure Profiling and Site-Resolved Elucidation of Ligand–Protein Interactions
Source: Anal Chem. 2026 Aug 3;98(32):23939–48. doi: 10.1021/acs.analchem.6c04688 (PMC13492042; doi:10.1021/acs.analchem.6c04688)
Supplement: Supplementary file 1 [file ac6c04688_si_001.pdf]

Supporting Information

HigH-ratiO partiaL proteolysiS with carriER proteome (HOLSER) Enables Global Structure Profiling and Site-resolved Elucidation of Ligand-Protein Interactions

Xuepei Zhang[1,2,3], Hassan Gharibi[1], Bohdana Sokolova[1], Zhaowei Meng[1,2,3], Hezheng Lyu[1], Amir Ata Saei[4], Massimiliano Gaetani[1,2,3], Roman A. Zubarev[1,3,5]**

[1] Division of Chemistry I, Department of Medical Biochemistry and Biophysics, Karolinska Institutet, SE-17 177 Stockholm, Sweden

[2] Chemical Proteomics Unit, Science for Life Laboratory (SciLifeLab), SE-17 177 Stockholm, Sweden

[3] Chemical Proteomics, Swedish National Infrastructure for Biological Mass Spectrometry (BioMS), SE-17 177 Stockholm, Sweden

[4] Department of Microbiology, Tumor and Cell Biology, Karolinska Institutet, SE-17 177 Stockholm, Sweden

[5] Department of Pharmacological & Technological Chemistry, I.M. Sechenov First Moscow State Medical University, Moscow, 119146, Russia

Table of Contents

Supplementary Figure S1 ·· S2

Supplementary Figure S2 ·· S3

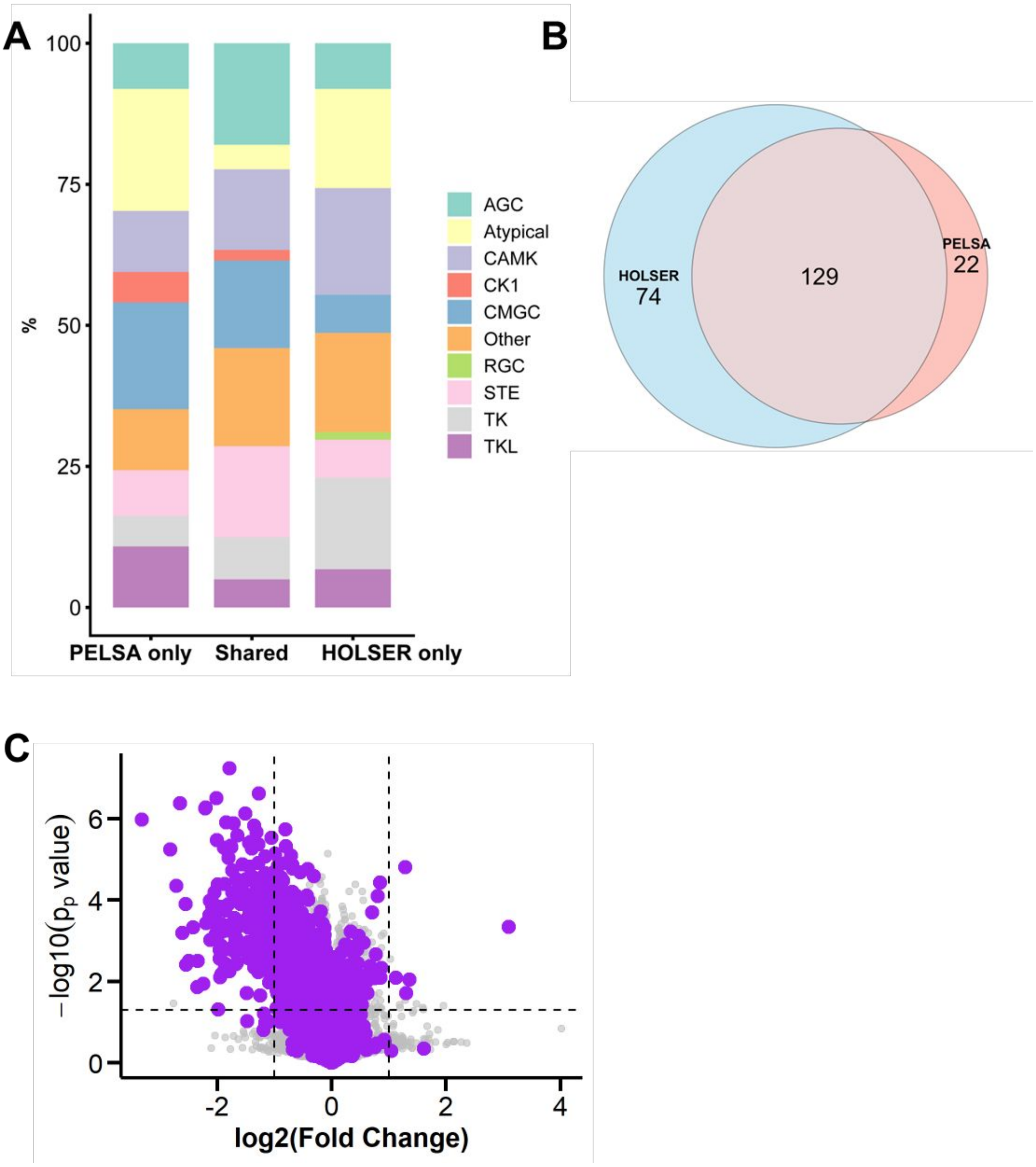

Supplementary Figure S1 | A, Distribution of kinase groups across PELSA-only, shared, and HOLSER-only targets. Bars show the number of kinases identified in each group. Kinases are classified into families according to UniProt annotations. The plot highlights kinases uniquely detected by PELSA (PELSA-only), those detected in both workflows (Shared), and those uniquely detected by HOLSER (HOLSER-only). **B,** Venn diagram illustrating the overlap in kinase targets (combined p-value < 0.05) on protein level in HOLSER and PELSA experiments. **C,** Volcano plot displaying peptide abundance changes in staurosporine -treated samples compared with DMSO controls (n=3 biological replicates). Peptides derived from kinases are highlighted in purple. Statistical significance was assessed by t-test.

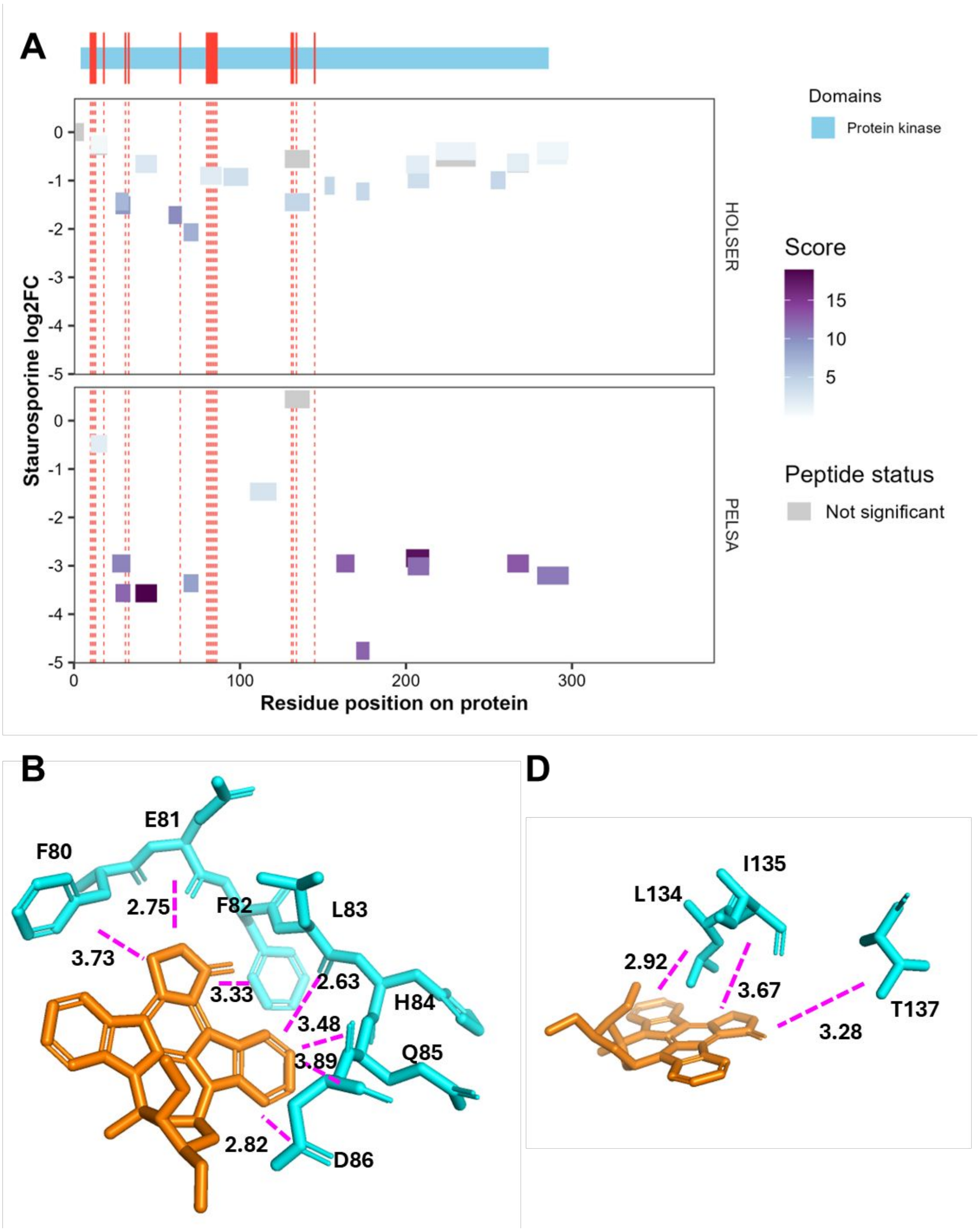
A
Domains
Protein kinase
Score
15
10
5
Peptide status
Not significant
Staurosporine log2FC
HOLSER
PELSA
Residue position on protein
0
100
200
300
0
-1
-2
-3
-4
-5
B
F80
E81
F82
L83
H84
Q85
D86
2.75
3.73
3.33
2.63
3.48
3.89
2.82
D
L134
I135
T137
2.92
3.67
3.28

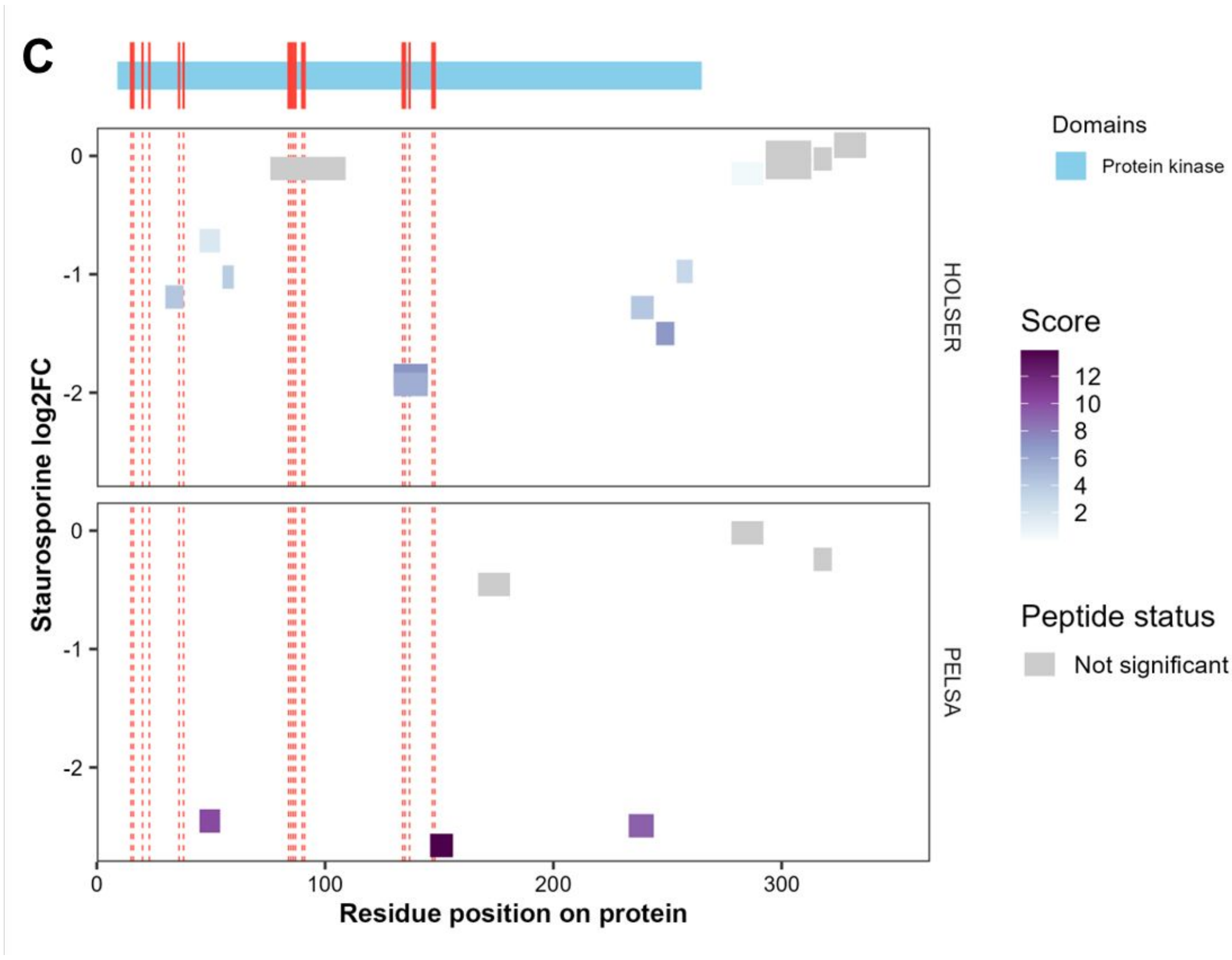

Supplementary Figure S2 | A, Mapping of CDK2-derived peptides identified in HOLSER and PELSA onto the protein sequence. Dashed lines indicate the known binding sites from PDB. **B,** Structural visualization of the CDK2–staurosporine complex based on the crystal structure (PDB ID: 1AQ1), rendered in PyMOL. **C**, Mapping of CHEK1. Dashed lines indicate the known binding sites from PDB. **D**, Structural visualization of the CHEK1–staurosporine complex based on the crystal structure (PDB ID: 1NVR), rendered in PyMOL.
